# From Black Box to Clear Insight: A Systematic Review of Human and Porcine Splenic Ex Vivo Perfusion Models

**DOI:** 10.7759/cureus.106263

**Published:** 2026-04-01

**Authors:** Tareq Al Saoudi, Edel Q Poopala Rayan, Pip Divall, Neama Alnabati, Wen Chung, John Isherwood, Deep Malde, Eyad Issa, Katrin Schilcher, Giuseppe Garcea, Marco R Oggioni, Ashley Dennison

**Affiliations:** 1 Hepato-Pancreato-Biliary Surgery, University Hospitals of Leicester NHS Trust, Leicester, GBR; 2 Genetics, Genomics and Cancer Sciences, University of Leicester, Leicester, GBR; 3 Clinical Library, University Hospitals of Leicester NHS Trust, Leicester , GBR

**Keywords:** basic research, human splenic ex vivo perfusion, normothermic machine perfusion, porcine splenic ex vivo perfusion, systematic review

## Abstract

The development of ex vivo perfusion models has significantly advanced our understanding of organ function, facilitating research into various health conditions. This review examines the use of porcine and human splenic ex vivo perfusion models and their contributions to biomedical research. We conducted a systematic literature review following Preferred Reporting Items for Systematic Review and Meta-Analysis Protocols (PRISMA-P) guidelines. Two independent reviewers screened titles, abstracts, and full texts to select studies on human and porcine spleen ex vivo perfusion models that met our inclusion criteria.

Nineteen studies using normothermic ex vivo perfusion on porcine spleens with different perfusion durations were reviewed. These studies demonstrated the adaptability and utility of these models in biomedical research. The earliest use of this model dates to 1966, with significant advancements over time. This progress has established the model as a crucial tool for studying bacterial pathogenesis. Both models have greatly enhanced our understanding of previously unknown mechanisms in haematology, parasitology, and microbiology. Currently, only two centres use the human splenic ex vivo perfusion model, with studies lasting up to six hours. Extending this duration could broaden the research scope, especially in microbiology and clinical immunology. Expanding the model's use could reduce reliance on animal models, aligning with the principles of the 3Rs: replacement, reduction, and refinement.

## Introduction and background

For a considerable period, the spleen was comparable to a “black box”, its secrets only hinted at through peripheral blood studies, with more invasive investigations impossible due to its anatomical position and fragility [1, 2]. Even today, the human spleen presents unique challenges to researchers for a number of well-recognised reasons. Firstly, the risk of intraperitoneal bleeding makes obtaining a biopsy a high-stakes procedure and one that is impossible to justify solely for research purposes [3]. Secondly, the marked differences in structure and function between human and animal spleens complicate and, for some species, invalidate comparative studies [4-6]. Lastly, post-mortem examinations, while useful, have their limitations, only revealing the late-stage manifestations of disease, and do not allow for the study of active splenic function [7].

Over the centuries, a myriad of functions have been attributed to the spleen. The central text of Rabbinic Judaism, the Talmud, refers to the spleen as the source of humour. The Greek word for 'black bile', *μέλαινα χολή* (melaina kholé), is the root of the English word 'melancholy', and black bile emanating from the spleen was believed to cause depression. Galen (c.130 AD - c.210 AD) was the first scientist and philosopher to assign specific parts of the soul to locations in the body, and he also introduced the concept of the spleen as a filter [8]. 

The spleen performs innate and adaptive (antibody and cell-mediated immune responses) functions of the immune system, facilitated by its organised morphology. It comprises two primary regions, the white pulp and the red pulp, each endowed with distinct roles. Within the white pulp resides a population of lymphocytes that assumes a pivotal role in the immune response. This role involves the production of antibodies and various immune cells that combat infection and disease. This process is facilitated through mechanisms such as antigen presentation by dendritic cells and macrophages, as well as the activation of T and B cells. In addition, the presence of receptors such as mannose receptors (which are well-characterised macrophage membrane lectins expressed on tissue macrophages throughout the body but not on circulating monocytes that play a role in the clearance of pathogens) aids in the spleen's filtration function, further underscoring its status as a lymphoid organ [9-10]. 

The red pulp has a very different function, serving as a critical blood filter, overseeing the health of the bloodstream. Its responsibilities include the removal of aged, damaged, or abnormal red blood cells, as well as any foreign substances or pathogens that may have entered the bloodstream. This filtration process is indispensable for the maintenance of a well-functioning circulatory system [11]. In addition to these vital immunological functions, the spleen serves as a reservoir for blood. It possesses the unique capability to expand or contract in response to the body's requirements; the contraction of the spleen on direct stimulation was first observed by Rudolph Wagner in the dog in 1849 [12], releasing stored blood during times of stress or injury to help maintain blood volume and pressure. Furthermore, it plays a significant role in platelet storage, releasing them into the circulation as needed to aid in haemostasis. During foetal development, it also serves as a crucial site for red blood cell production, a function that can be reactivated in pathological conditions such as thalassaemia [11-14]. 

The spleen is unusual, being an organ that is frequently removed to treat benign and malignant conditions affecting the left part of the pancreas. This is primarily carried out for surgical and oncological reasons due to the spleen's close proximity to the tail of the pancreas and its fragile nature, which makes preservation technically difficult. Removal improves the clearance margin of tumours, aids any required lymphadenectomy to reduce the risk of tumour spread and local recurrence, and is not infrequently required to aid access and visualisation, rather than being linked to any inherent spleen abnormalities [15]. This unique property offers the opportunity to use the resected human spleens for ex vivo perfusion without compromising the patient’s clinical management or posing any ethical dilemma.

Animal models have been employed for over 2000 years to improve our understanding of behaviour, health, and biology [16]. Whether in vivo or in vitro, they allow investigators to control variables during perfusion and, in ex vivo models, can isolate organs from all metabolic and physiological influences, facilitating the examination of changes consequent upon the alteration of a single parameter (physiological or pharmacological) [17]. Various animal organs, including the spleen, have been studied to better understand important human physiological, immunological, and pathological processes and disease resistance [18]. Although there are reports of the use of spleens from murine and other small animals, the porcine spleen is favoured by researchers. It closely mirrors the human organ, boasting high neutrophil counts, similar microanatomy, and comparable splenic and lymph node macrophage subpopulations, T-cell subpopulations, and cytokine profiles. In addition, pigs and humans share more than 80% of their immune parameters [19]. 

Ex vivo perfusion is a technique used to preserve organs outside the body for extended periods while maintaining their anatomical and physiological integrity. In 1935, Alexis Carrel and Charles Lindbergh reported the first successful ex vivo perfusion of organs, specifically cat thyroid glands and ovaries, which were perfused for over 20 days [20]. Since then, the ex vivo perfusion model has dramatically changed from hypothermic to normothermic perfusion through a number of iterations facilitated by technical advances, particularly from groups involved in organ preservation and transplantation studies. Ex vivo perfusion enables the preservation of organ function for significant periods of time, which provides an excellent model for laboratory experiments and clinical trials [21]. Additionally, it serves as a valuable method for evaluating organs prior to transplantation and even "reconditioning" them, thereby increasing the number of viable organs available for transplantation that would otherwise be rejected by transplant teams [22, 23].

In this narrative review, we will summarise the literature on ex vivo perfusion experiments involving both human and porcine spleens. Our focus will be on the development, current approaches, technical aspects, challenges, and applications of data from available studies.

## Review

Methods 

A systematic literature review was performed according to the Preferred Reporting Items for Systematic Reviews and Meta-Analyses for Protocols (PRISMA-P) [24]. We performed a search of all the published literature, focusing on qualitative and quantitative studies published in English from 1946 until January 2025. To conduct the search, we utilised the databases Medical Literature Analysis and Retrieval System Online (MEDLINE), Excerpta Medica database (Embase), and Scopus, using combinations of keywords including "spleen or splenic", "ex-vivo or ex vivo", "ex situ", "extracorporeal", "perfus*", "perfusion" to cast a wide net to identify relevant literature on ex vivo spleen perfusion. Additionally, we manually reviewed the reference lists of included studies and review articles to ensure complete capture of any relevant research. The primary outcome of interest was to understand the current application and use of human and porcine spleen ex vivo perfusion, the technical aspects of the perfusion process, and the associated clinical implications. 

Two independent reviewers (TA and EP) employed a two-stage method to conduct the study screening independently and evaluated the titles and abstracts of the identified studies to determine their potential relevance to human and porcine spleen ex vivo perfusion. At the first stage, titles and abstracts were screened to exclude obviously ineligible studies. At the second stage, the full text was read, looking for further eligible studies. Disagreements were resolved via consensus and discussion with the chief author (ARD). Subsequently, we obtained and assessed the full texts of studies that met the inclusion criteria. 

These criteria encompassed studies that provided original data pertinent to human and porcine spleen ex vivo perfusion, elucidated the technical aspects of perfusion machinery, or reported clinical outcomes and experimental findings associated with this subject matter. Studies exclusively focused on organs other than the human and the porcine were excluded. Inclusion criteria included articles in the English language, full-length research articles, and studies specifically addressing human and porcine spleen ex vivo perfusion. Exclusion criteria included abstracts and presentations, review articles, and investigations conducted with models other than human or porcine.

**Figure 1:**
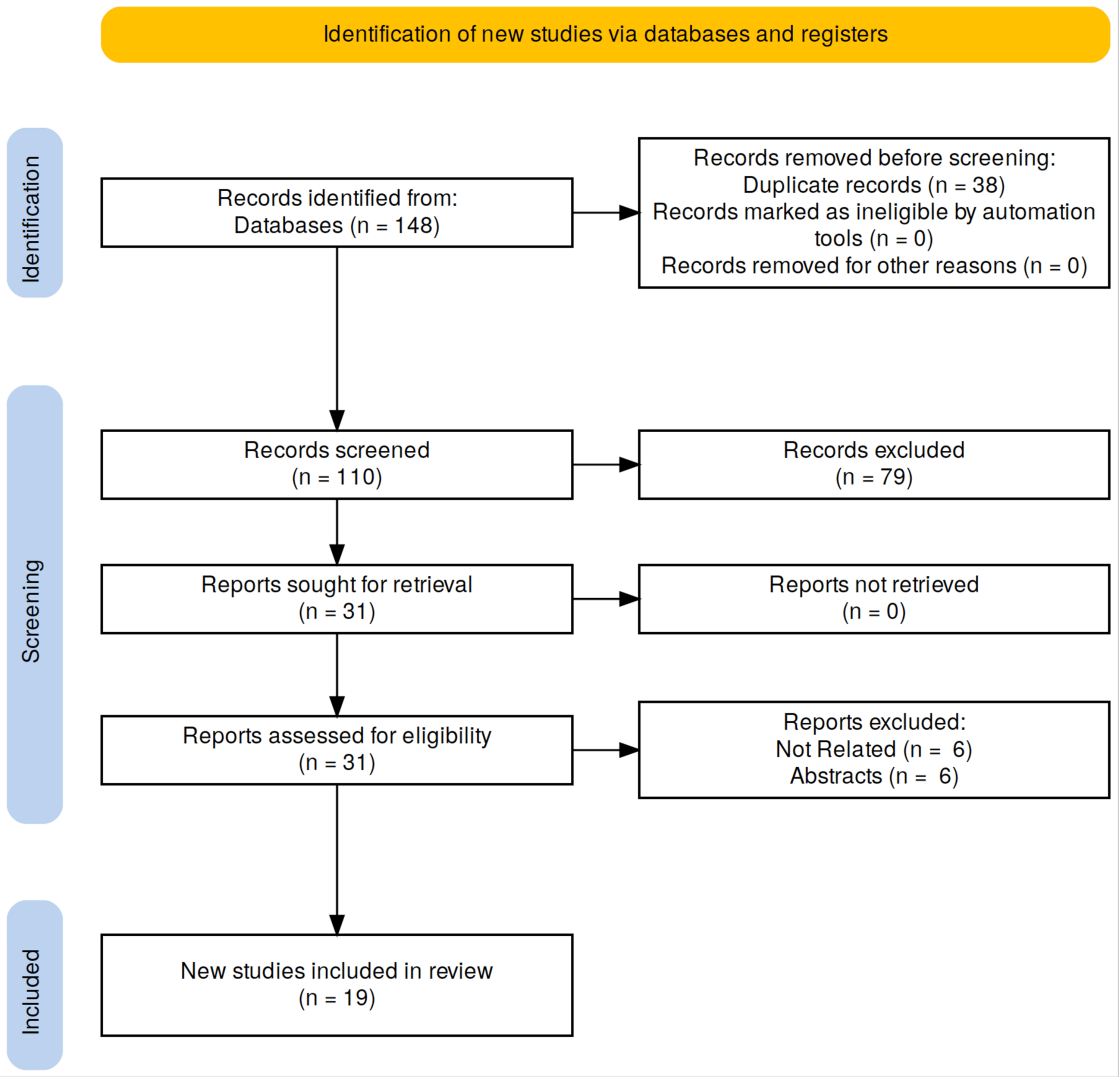
PRISMA flow diagram describing the literature search and article selection process. PRISMA: Preferred Reporting Items for Systematic Reviews and Meta-Analyses

Data extraction was performed by the same reviewers using a pre-designed form. The form included author information, publication year, study design, sample size, the type of ex vivo perfusion machine used, and details of the perfusion protocol (including preservation solution, duration, and temperature). As this review is narrative, no quantitative synthesis or meta-analysis was conducted. Instead, we qualitatively assessed and summarised the findings from each study, aiming to identify patterns across them. Figure 1 shows the PRISMA flow diagram highlighting the literature search process, number of articles and inclusion and exclusion criteria. 

Review

Human Splenic Ex Vivo Model

Nine articles that aligned with our inclusion criteria discussed the human splenic ex vivo perfusion model. Notably, all these studies conducted their experiments under normothermic conditions, utilising spleens obtained during distal pancreatectomy and splenectomy procedures. Additionally, macroscopic and microscopic examinations confirmed that all of the spleens were normal. It's important to highlight that in all cases, the spleen perfusion lasted for four to six hours, although there were significant variations in terms of the cold flush technique employed and the type of perfusate used, which were contingent on the specific research centre and the scientific objectives of the respective studies.

Development and proof of concept: Buffet et al., in 2006, were the first group to utilise the ex vivo perfusion model for studying the human spleen [25]. They developed their model based on the ex vivo perfusion model of pig spleen, taking advantage of the similarities in size and vascular anatomy between pigs and humans [18]. They were interested in the response to malaria in Malawians who had previously had a splenectomy, recognising the crucial role that the spleen plays in the pathogenesis of malaria (evidenced clinically by the increased rate of fever and parasitaemia in patients following splenectomy) [26]. They also considered the fact that drug-exposed parasites, particularly following treatment with artesunate (one of a group of drugs derived from "qinghao", or the sweet woodworm plant), are primarily cleared by the spleen in patients with *Plasmodium falciparum* malaria, making it a functional assessment parameter for the spleen during ex vivo perfusion [27]. 

To validate their model, Buffet et al. prepared ring-stage parasites and added artesunate to the preparation eight to 12 hours before introducing them to the perfusion circuit. The ring stage represents the initial stage of the *Plasmodium falciparum* erythrocytic cycle, characterised by the development of merozoites into a ring-shaped form. This stage plays a crucial role in the pathogenesis of malaria, as it contributes to parasite development and immune evasion and serves as a target for antimalarial drugs [26, 27]. The count of artesunate-exposed infected red blood cells (Art-iRBCs) decreased within 10-30 minutes after establishing the perfusion model, with a half-life of 17-18 minutes and an overall clearance time of 120 minutes, after which 95% of the parasites could no longer be detected. Additionally, they examined another functional parameter of the spleen using this model, known as pitting. Pitting is a physiological process where the spleen removes inclusion bodies containing dead or foreign material from erythrocytes as they pass through narrow splenic venous sinuses [28]. This process has been used to assess splenic function, as the presence of more than 2% of circulating red blood cells (RBCs) with pitted features in patients indicates a higher risk of overwhelming sepsis [29]. In this model, immunofluorescence assays revealed an increase in the number of pitted Art-iRBCs, indicating an active pitting process. An ultrasound examination of the spleen demonstrated a homogeneous texture with no significant areas of ischaemia or air bubbles, and Doppler ultrasonography indicated excellent perfusion. A remarkable finding was that despite the red pulp comprising 75% of the splenic volume, more than 90% of the Art-iRBCs were retained within it, highlighting the distinct activity and compartmentalisation. This proof-of-concept study was important, as it provided evidence that the spleen can be studied while being perfused on an ex vivo circuit and that this technology maintained its physiological function. Consequently, it established ex vivo perfusion as a viable model for translational clinical research and demonstrated for the first time the functional differences of the various zones of the human spleen.

Disease dynamics in malaria infection: Safeukui et al. in 2008 utilised a combination of in vivo and ex vivo models to investigate malaria pathophysiology in the spleen and gain further insights into the ring stage population [30]. Prior to this study, it was generally believed that young ring-infected erythrocytes circulated in the bloodstream, while maturely infected cells were retained by the spleen [31]. 

While this study did confirm that mature infected cells were indeed retained by the spleen, it also demonstrated that this was the case for 50% of the young ring-infected cells [30]. Furthermore, with each passage through the ex vivo perfusion circuit, 10% of the young ring-infected cells were retained in the spleen. This finding led for the first time to the identification of two distinct populations of cells within the ring stage. The first population exhibited deformability and was able to pass through the spleen, continuing to circulate in the blood. Conversely, the second population consisted of less deformable cells with elongated axes, unable to bypass the narrow spaces within the spleen. These findings, combined with measurements of RBC elongation, which indicated mildly elevated deformability, led to a paradigm shift in the understanding of the kinetics of erythrocytes within the spleen and malarial pathogenesis. 

To determine the retention rate of normal uninfected RBCs and *Plasmodium falciparum*-iRBCs at different stages, namely ring-infected and trophozoite-iRBCs, Safeukui et al. [32] employed ex vivo perfusion of human spleens and a microfiltration device based on microbeads, as described by Deplaine et al. [33]. They utilised ImageStream technology for the analysis of the morphology of iRBCs and cellular dimensions. The study revealed that ring-iRBCs displayed reduced membrane surface area and increased sphericity, resulting in decreased deformability and an elevated retention rate. The estimated surface area loss for the retained ring-iRBCs was approximately 17%, which aligned with the findings of lysophosphatidylcholine (LPC)-exposed RBC retention as described by Safeukui et al. [34], findings which have significant implications. Firstly, they contribute to the modelling of infection kinetics, enabling a better understanding of the parasite load and analysis of risk factors associated with severe clinical forms of malaria. Secondly, they are important in the diagnosis of spleen functionality and offer insights into potential adjuvant therapies targeting ring-iRBCs, thereby influencing drug treatment strategies for malaria.

In 2016, Diakité et al. conducted a study to explore the protective effect of the sickle cell trait on severe malaria episodes and lower levels of parasitaemia observed in children with this trait compared to those with normal haemoglobin [35, 36]. It was hypothesised that splenic-dependent removal of ring-infected cells might be the underlying cause, as sickle cell trait RBCs are known to be less deformable than normal RBCs and are presumed to have increased retention in the spleen [37]. Additionally, it has been observed that ring-iRBCs sickle faster than uninfected haemoglobin S (HbS) upon treatment with reducing agents [38]. These hypotheses were tested using an ex vivo perfusion model with donor blood from both France and Mali, along with a microfiltration device as an adjunct. Despite the logical assumption, the study found that HbS-iRBCs were retained in the spleen at a similar rate to normal Hb-infected cells in both the ex vivo perfusion model and the microfiltration device. This demonstrated that sickle cell trait does not suppress parasitaemia by enhancing mechanical retention. However, the study also revealed that mature RBCs infected with malaria were efficiently retained in the spleen, despite the lack of cytoadherence mechanisms due to impaired function with HbS [39]. This emphasised the increased efficiency of mechanical retention by the spleen and provides a potential explanation for the protective effect of sickle cell trait in malaria infection.

RBC disorders and blood transfusion: The spleen plays a crucial role in retaining less deformable RBCs in a number of pathological conditions, including hereditary spherocytosis and malaria [40]. Extensive entrapment of RBCs in the spleen can lead to anaemia, while a decrease in this activity has been associated with complications [41, 42]. This activity is also important in stored blood that exceeds three weeks, as RBC survival is influenced by storage time and has been linked to transfusion-associated complications [43, 44]. Deplaine et al. analysed the anatomical structure of the spleen with the aim of creating a model using microbeads to mimic the inter-endothelial slits, the narrowest circulatory pathway in the human spleen, in an ex vivo perfusion model [33]. The researchers developed a device containing a mixture of microbeads with diameters ranging from 5 to 25 μm to replicate the mechanical sensing of RBCs in the splenic microcirculation. They observed that heated RBCs, RBCs infected with *Plasmodium falciparum* (Pf-RBCs), and RBCs from patients with hereditary spherocytosis (HS-RBCs) were retained in the microbead layer without undergoing haemolysis, as indicated by stable low levels of lactate dehydrogenase. The retention rates of Pf-RBCs, HS-RBCs, and heated RBCs in the microbeads were similar to those observed in isolated perfused human spleens. 

These findings confirmed the importance of mechanical sensing of RBCs by the human spleen and concluded that the inter-endothelial slits of the spleen and microbeads force RBCs to deform similarly. This is an important finding, as it demonstrates that the mechanical properties of the spleen can be replicated in vitro, providing a valuable model considering the limited access to human spleens. Additionally, it offers a simple and inexpensive means for diagnosing and understanding various RBC disorders and provides proof of concept that this model can identify and separate rigid RBCs in blood that has been stored for long periods. Further confirmation came from the demonstration that in the samples studied, the deformability of the RBCs was restored to normal levels, offering a potential method to clear effete RBCs to improve transfusion yield and reduce complications associated with transfusions. 

Safeukui et al. performed a study to investigate splenic entrapment of diseased RBCs, particularly in the context of hereditary spherocytosis [34]. Hereditary spherocytosis is an autosomal dominant inherited disease characterised by a deficiency in membrane proteins, primarily Spectrin. This deficiency leads to a change in the shape of RBCs, transitioning them from a biconcave discoid shape, which allows for significant deformability while maintaining a constant surface area, to spherocytes that have limited deformability [42]. Prior to this study, there was a lack of quantitative data regarding the relationship between the degree of surface area loss and the degree of splenic entrapment. Safeukui et al. took advantage of a unique feature of LPC, which induces dose-dependent surface area loss by exclusively accumulating in the external leaflet of the RBC lipid bilayer. In the ex vivo blood-perfused spleen model, LPC-treated and untreated RBCs were labelled using lipophilic fluorescent probes, and RBC deformability, osmotic fragility, and electron microscopy measurements were conducted. High doses of LPC produced perfectly spherical RBCs with the predicted consequent surface area loss, and the spleen was capable of clearing LPC-treated RBCs with a mean half-time of 3.5 minutes. The degree of surface area loss required for the spleen to clear 90% of the RBCs is 18%, which corresponds to a decrease of over 27% in the surface area-to-volume ratio. Studies using this important ex vivo splenic perfusion model demonstrate that the loss of surface area serves as a predictor of splenic entrapment. This finding has clinical implications in assessing splenic function and conducting drug screening to identify substances that may potentially induce haemolytic anaemia [43, 44].

The deformability of RBCs is related to three separate parameters: the cell surface area-to-volume (S/V) ratio, intracellular viscosity, and membrane viscoelasticity [45]. In severe haemolytic anaemia, abnormalities in one or more of these parameters can cause splenic entrapment and subsequent anaemia [46]. While the S/V ratio has been previously studied by Safeukui et al. and shown to be a predictor of splenic entrapment, the impact of reduced deformability on the entrapment of RBCs in the spleen remained unclear. In an attempt to answer this question, Safeukui et al. in 2018 [47] induced reduced membrane deformability by treating RBCs with diamide, a sulfhydryl oxidant that promotes the formation of inter- and intramolecular disulfide bonds, resulting in increased membrane rigidity. The level of membrane rigidity increased with the dose of diamide [48], and using an ex vivo splenic perfusion model and a microfiltration device, they were able to demonstrate that treated RBCs were rapidly retained in the spleen in a dose-dependent manner. However, this relationship was more modest in the microfiltration device, and RBCs with the highest degree of reduced membrane deformity were not retained, suggesting that mechanical entrapment may not be the primary mechanism for treated RBCs. This finding was further supported by the observation that RBCs from patients with Southeast Asian ovalocytosis, an inherited disorder characterised by a significant decrease in membrane deformability, circulated freely in the vascular bed without significant sequestration by the spleen [49]. These findings provided support for the hypothesis that ligand adhesion molecules, rather than mechanical entrapment, underlie the mechanism of splenic entrapment in disorders characterised by decreased membrane deformability.

Although the impact of using fresher blood compared to standard stored blood on mortality has been shown to be insignificant [50, 51], its effect on morbidity, particularly in cases of massive transfusion and chronic transfusion, is not yet fully understood. Cold storage of blood is known to be associated with the appearance of altered morphology and smaller RBCs, referred to as storage-induced microerythrocytes (SMEs). These SMEs include type III echinocytes, spheroechinocytes, and spherocytes and can comprise up to 24% of the RBC population in blood stored for 42 days [52, 53].

In a study conducted by Roussel et al. in 2021 [54], an ex vivo splenic model was used to investigate the retention rate of SMEs and their correlation with transfusion recovery. The researchers found that SMEs accumulated in a manner that correlated with transfusion recovery, and the percentage of SMEs increased as the storage period of blood increased. This suggests that the percentage of SMEs could potentially serve as a powerful predictor of RBC storage quality and transfusion recovery, as well as for assessing new manufacturing processes such as hypoxic storage [55].

Role of the spleen in pneumococcal bacteraemia: Carreno et al. conducted a study investigating the role of splenic macrophages in pneumonia-associated bacteraemia [56]. It has long been assumed that bacteraemia in severe community-acquired pneumonia originates solely from pneumococci entering the bloodstream from the lungs. By employing three different models, including a human ex vivo splenic perfusion model, the researchers were able to demonstrate that the spleen may play a significant role in the pathogenesis of bacteraemia. Carreno et al. were able to demonstrate that pneumococci tend to cluster within splenic macrophages. When these bacteria were treated with sub-inhibitory concentrations of azithromycin, which has no direct effect on pneumonia itself but concentrates in the splenic macrophages, no bacteria were detected in the spleen or blood, and most importantly, there were no signs of disease. This finding indicates that the bacterial load in the spleen, rather than the lungs, correlates with the occurrence of bacteraemia. The results also support the hypothesis that the spleen, not the lungs, serves as the primary source of bacteria during systemic infection associated with pneumococcal pneumonia and provides a basis for utilising combination therapies, including macrolides, in the treatment of severe community-acquired pneumococcal pneumonia [57]. 

Table 1 provides a summary of the diverse characteristics of the ex vivo perfusion process described in each of the selected studies.

**Table 1:** Key findings of the included studies on human splenic ex vivo perfusion models *Cold Krebs–albumin solution: (25 mmol sodium bicarbonate (NaHCO₃), 118 mmol sodium chloride (NaCl), 4.7 mmol potassium chloride (KCl), 1.2 mmol magnesium sulfate heptahydrate (MgSO₄·7H₂O), 1.2 mmol sodium dihydrogen phosphate (NaH₂PO₄), 1.2 mmol calcium chloride dihydrate (CaCl₂·2H₂O), 7 mmol glucose (C₆H₁₂O₆), and 5 g human serum albumin (Albumax), in 1 L sterile water); RBC: red blood cells; RPMI: Roswell Park Memorial Institute

Number	Year	Authors	Number of perfusions	Spleen macro- and microscopic morphology	Warm ischaemia time	Flush used	Perfusate type	Cold ischemia time	Perfusion temperature (°C)	Operation
1	2006	Buffet et al. [25]	6	Normal spleens, both macro- and microscopically	30-90 mins (according to surgical factors)	Cold Krebs -albumin*	Krebs-albumin/ RBCs	60-90 mins	37 °C	Distal pancreatectomy and splenectomy
2	2008	Safeukui et al. [30]	10	Normal spleens, both macro- and microscopically	30-90 mins (according to surgical factors)	Cold Krebs -albumin	Krebs-albumin/ RBCs	60-90 mins	37 °C	Distal pancreatectomy and splenectomy
3	2011	Deplaine et al. [33]	-	-	-	Cold Krebs -albumin	-	-	-	Distal pancreatectomy and Splenectomy
4	2012	Safeukui et al. [34]	9	Normal spleens, both macro- and microscopically	30 mins	Cold RPMI 1640 -albumin	Washed blood		37 °C	Distal pancreatectomy and splenectomy
5	2013	Safeukui et al. [32]	-	-	-	-	-		-	-
6	2016	Diakité et al. [35]	-	Normal spleens, both macro- and microscopically	30-90 mins (according to surgical factors)	Cold RPMI 1640 -albumin	Mixture of RBCs suspension	60-90 mins	37 °C	Distal pancreatectomy and splenectomy
7	2018	Safeukui et al. [47]	-	Normal spleens, both macro- and microscopically	30-60 mins (according to surgical factor)	Cold RPMI 1640 -albumin	Krebs-albumin with RBCs	60-90 mins	37 °C	Distal pancreatectomy and splenectomy
8	2021	Roussel et al. [54]	-	Normal spleens, both macro- and microscopically	-	Cold Krebs -albumin	Kerbs-albumin/ stored RBCs	-	37 °C	Distal pancreatectomy and splenectomy
9	2021	Carreno et al. [56]	12	Normal spleens, both macro- and microscopically	-	Soltran preservative solution	Hemopure		37 °C	Distal pancreatectomy and splenectomy

Porcine Splenic Ex Vivo Model

From our literature search, we identified 10 articles fitting the pre-determined criteria that discussed the porcine splenic ex vivo perfusion model. It is notable that although there were variations in the cold flush and perfusate details depending on the study’s specific aim and scientific application, every study performed experiments under normothermic conditions. Perfusion durations also varied, ranging from an hour to over a week. 

Development and proof of concept: In 1948, Hechter et al. developed the concept of animal spleen perfusion in rabbits using autologous blood. Although the lymphocyte count remained stable over six hours, histology revealed lymphocyte depletion, leading them to conclude that spleen destruction had occurred. Nearly two decades after Hechter's work, in 1966, Boxall et al. achieved the first successful ex vivo perfusion of a porcine spleen [58]. They examined splenic lymphocyte production using lymphocyte counts and assessed phagocytic activity by measuring the clearance of microcurie colloidal gold (Au¹⁹⁸), which is ingested by the phagocytes and can be traced and identified by its radioactive properties [59]. Of the 104 porcine spleens examined, roughly half were perfused with autologous (porcine) blood, while the remainder used heterologous (human) blood. The experiments demonstrated an increase in lymphocyte numbers, more pronounced in the spleens perfused with human blood. The colloidal gold extraction ratio was 25% for the autologous group and, notably, 42% for the heterologous group. The authors were not able to explain the enhanced function of the porcine spleen with heterologous blood but did note that heterologous blood led to an increase in vascular resistance, while oxygen utilisation was superior with autologous blood. These studies were the initial proof of concept showing that the function of the porcine spleen could be maintained using an ex vivo perfusion model. Furthermore, the porcine spleen's function was preserved when human blood was used as the perfusion medium, presenting significant potential for subsequent clinical immunological studies. 

In a subsequent study in 1967, Boxall et al. reported the ex vivo perfusion of 65 porcine spleens [60]. Using a similar approach to their previous experiments, they perfused the spleens with both autologous and heterologous blood. Lymphocyte count measurements revealed a 76% increase by the fourth hour of perfusion, with a significant 196% surge in the first hour. The authors attributed this phenomenon to the spleen being flushed out with perfusate and the subsequent production of new lymphocytes. This production increase was consistent for both blood types. They also examined vascular resistance, oxygen utilisation, and glucose utilisation, and conducted haematological studies. While oxygen utilisation, glucose composition, and haematological results were consistent across both perfusion groups, vascular resistance was two to three times higher in the heterologous group and was unaffected by vasoactive agents or re-heparinisation. They emphasised the importance of using fresh blood as opposed to old, stored blood, as the latter resulted in reduced perfusion rates. The study confirmed that the spleen's function remains intact when perfused with human blood, suggesting a potential use for further immunological studies and clinical immunotherapy. 

Moore et al. assessed the ex vivo perfused spleen for its potential as a source of immunologically competent lymphocytes and its filtering capability for normal leucocytes and circulating leukaemic cells [61]. They perfused 16 porcine spleens using various blood types, including heparinised human blood, heparinised porcine blood, blood from a patient with chronic myeloid leukaemia (CML), and blood from pigs previously injected with sheep RBCs (sRBCs). They measured the leucocyte counts and identified antibody-producing lymphocytes. Experiments were conducted both with and without the spleen in the circuit. Without the spleen, leukocyte counts remained unchanged; however, with the spleen, lymphocyte counts rose by about 3,000 cells per ml³/hour regardless of whether human or porcine blood was used. Notably, the count of leukaemic cells dropped significantly during ex vivo splenic perfusion, from 90% initially to just 2% after one hour. This study demonstrated that the pig spleen, when perfused with various blood types, can reduce antigen load through its leukopenic effects. It also produces lymphocytes active against specific antigens, suggesting its potential therapeutic value in conditions like leukaemia or other neoplasms with circulating tumour cells.

Pushing the limits:* *Prior to 1968, porcine ex vivo splenic perfusion was limited to eight hours. Atkins et al. extended this by using the ex vivo porcine perfusion model to test a single membrane oxygenator device designed for prolonged organ perfusion or preservation before transplantation [62]. They perfused forty porcine spleens for up to seven days at 37°C using this oxygenator. Its key benefit was its large gas transfer surface relative to its small priming volume, eliminating gas-perfusate interference and enabling oxygen tension regulation. Two years later, Atkins et al. detailed their technique for extended ex vivo perfusion, positioning it as a model for potential antibody production [63]. They emphasised the significance of using a sanguineous perfusate for maintaining the spleen over extended periods.

Building on the work by Atkins et al. demonstrating the feasibility of a prolonged perfusion model, in 1969, Ruble et al. optimised oxygen tension and perfusion rates to enhance the metabolic function of the ex vivo perfused porcine spleen, using lactate production, oxygen utilisation, and glucose consumption as surrogate markers of successful preservation [64]. They tested various oxygen saturations and pressures with a constant flow. Their findings demonstrated that an oxygen pressure of 251 to 350 mmHg optimised aerobic glucose metabolism, leading to efficient adenosine triphosphate production (ATP). Additionally, a flow rate of 1-3 ml/g of splenic weight yielded optimal metabolic activity, while rates below 0.5 ml/gm inhibited metabolism (using glucose metabolism as a benchmark).

In 1970, Atkins et al. performed further studies utilising the ex vivo porcine model to investigate the spleen's capacity to produce antibodies when exposed to an antigen [65]. They perfused 27 porcine spleens using the oxygenator they previously developed, running each experiment for up to a week [62]. Two pigs were pre-injected with a 50% suspension of sRBCs three days before splenectomy. Four hours into the perfusion, sRBC antigen was introduced, with six spleens left unchallenged as controls. After perfusing for one to five days without changing the medium, the perfusate was collected. They observed increasing amounts of antibody-producing lymphocytes in both the spleen and perfusate. This research represented the first demonstration that an isolated perfused organ can generate antibodies after antigenic exposure.

Bacterial sepsis pathogenesis: Until very recently, the mechanisms by which *Streptococcus pneumonie* induces septicaemia leading to clinical sepsis were unclear, especially in patients without evident risk factors. Ercoli et al. employed an ex vivo porcine splenic model to investigate the role of splenic macrophages in *Streptococcus pneumoniae* septicaemia [66], notably sourcing animals from the food chain, obviating the need for live animal studies. Their studies demonstrated that shortly after infection, pneumococci are captured by red pulp macrophages and by CD169+ macrophages in the perfused spleen. This environment allows bacterial replication, seemingly shielding the bacteria from the host's immune defences and serving as reservoirs for bacteria which subsequently re-enter the bloodstream, leading to septicaemia and sepsis. This discovery may explain the superior efficacy of macrolides over beta-lactams, the standard therapy for pneumococcal pneumonia, in preventing septicaemia [56]. Understanding this early intracellular phase, where bacterial numbers are limited, offers new avenues for targeted treatments and effective clinical management.

In 2019, Chung et al. employed a porcine model to examine early splenic responses following infection but preceding bacterial sepsis [67]. Their ex vivo porcine splenic perfusion model extended the work of Ercoli et al. by introducing both Gram-negative and positive bacteria to the perfusion circuit [66]. They found that while Gram-negative bacteria survived in porcine blood without proliferating, Gram-positive species survived and replicated. This difference was attributed to the superior complement resistance of Gram-positive bacteria [68]. They subsequently focused on *Streptococcus pneumoniae* due to the spleen’s significant role in its infectious pathogenesis [69]. In the course of infection, there was an apparent increase in neutrophil numbers, suggesting the spleen's heightened and localised defence response, distinct from the systemic circulation. Similarly, cytokine levels increased in samples that were infected compared with controls. Microscopic analysis confirmed the preservation of splenic micro- and macro-architecture. This study demonstrated the spleen's resilience and functionality during ex vivo perfusion, highlighting its potential for studying early bacterial infections and evaluating the efficacy of antimicrobial treatment and regimens.

Wanford et al. used an ex vivo porcine spleen and liver co-perfusion model to examine the role of macrophages in the pathogenesis of *Klebsiella pneumoniae* [70]. This bacterium has been linked to liver abscess formation, becoming a leading cause in Asia and the USA [71]. Despite its prevalence, its pathogenesis and the roles of different macrophages in its clearance remained unclear [72]. The study examined two strains, hypervirulent *Klebsiella pneumoniae *(hvKP) and non-hvKP. They found that the hvKP strain led to a localised influx of neutrophils with subsequent replication within macrophages and the formation of micro-abscesses, whereas the non-hvKP strain was effectively cleared. The findings suggest that the hvKP strain resists Kupffer cell-mediated clearance, which delays neutrophil clearance, potentially causing hepatic abscesses, with implications for antibiotic treatments and the choice of appropriate agents. Table 2 outlines the distinct characteristics of the ex vivo perfusion techniques detailed in these studies.

**Table 2:** Key findings of the included studies on porcine splenic ex vivo perfusion models

Number	Year	Authors	Number of perfusions	Flush used	Perfusate type	Perfusion temperature (°C)	Perfusion time
1	1966	Boxall et al. [58]	104	2L heparinised Ringer’s lactate	Porcine blood and human blood	-	Up to 8 hours
2	1967	Boxall et al. [60]	65	2-3L heparinised Ringer’s lactate	Porcine blood and human blood	37 °C	6 hours
3	1968	Moore et al. [61]	16	-	Porcine blood, human blood, and blood from chronic myeloid leukaemia patients	-	1 hour
4	1968	Atkins et al. [62]	40	-	-	37 °C	Up to 7 days
5	1969	Ruble et al. [64]	8	2L of 0.9% sodium chloride with 10,000 units of heparin, 60 mg procaine hydrochloride and 7 mEq of sodium bicarbonate/litre	McCoy's 5A medium containing glucose, 3 mg/ml; penicillin G, potassium, 0.5 mg/ml; streptomycin sulphate, 0.5 ug ml; and neomycin sulphate, 0.1 ng/mi was used. Pooled pig serum was added to a 10% concentration, and porcine regular insulin to 0.25 units/mL.	37 °C	-
6	1970	Atkins et al. [63]	-	2L of 0.9% sodium chloride with 10,000 units of heparin, 60 mg procaine hydrochloride and 7 mEq of sodium bicarbonate/litre	600 ml of medium 199 with 10% foetal calf serum, to which penicillin, neomycin, and glucose were added.	37 °C	Up to 7 days
7	1970	Atkins et al. [65]	27	2L of 0.9% sodium chloride with 10,000 units of heparin, 60 mg procaine hydrochloride and 7 mEq of sodium bicarbonate/litre	600 ml of medium 199 with 10% foetal calf serum, to which penicillin, neomycin, and glucose were added.	37 °C	Up to 7 days
8	2018	Ercoli et al. [66]	-	Saline solution containing heparin and human urokinase	1L of autologous porcine blood, containing nalidixic acid (10 mg/L) and colistin (5 mg/L), glucose (5 ml/h), 500 μg epoprostenol sodium (vasodilatation), and 5000 IU heparin (microclot prevention; 1500 units/hr)	37 °C	6 hours
9	2019	Chung et al. [67]	7	1L of Soltran preservation solution (Baxters, UK) containing 5,000 IU of heparin and 10,000 IU of human urokinase	1L of porcine heparinised blood	37 °C	6 hours
10	2021	Wanford et al. [70]	3		2L heparinised autologous pig's blood	37 °C	6 hours

Limitations

This review is subject to several limitations inherent to the available literature and the narrative review methodology. First, although we conducted a comprehensive literature search, the scarcity of studies utilising the human splenic ex vivo perfusion model limits the generalisability of our conclusions. Furthermore, heterogeneity in perfusion techniques, perfusate composition, and methodological reporting across studies complicates direct comparison and synthesis of findings. The absence of quantitative meta-analysis and reliance on qualitative assessment introduces a degree of subjectivity. Lastly, publication bias and the exclusion of non-English studies may have led to the omission of relevant research.

## Conclusions

Both human and porcine ex vivo splenic perfusion models have greatly enhanced our understanding of previously unknown mechanisms in haematology, parasitology, and microbiology. These studies have revealed unrecognised interactions and functions of the spleen, clarified poorly understood illnesses, and highlighted the potential for improved treatments of widespread and often fatal diseases. They also provide valuable insights into the spleen's role in various health conditions.

Currently, only two centres have access to the human splenic ex vivo perfusion model, and there is a significant lack of detailed data on the technical aspects of this research tool. Furthermore, the duration of these studies is typically limited to a maximum of six hours. Extending this duration could generate more data and broaden the scope of pathologies that can be investigated, particularly in the fields of microbiology and clinical immunology.

Moreover, expanding the use of the human splenic ex vivo perfusion model could address the limitations associated with animal models, despite their anatomical, physiological, biochemical, and immunological similarities to humans. Crucially, it could also reduce the need for animal experiments, aligning research practices with the principles of the 3Rs: replacement, reduction, and refinement.
